# Formulation of Thermo-Sensitive In Situ Gels Loaded with Dual Spectrum Antibiotics of Azithromycin and Ofloxacin

**DOI:** 10.3390/polym16212954

**Published:** 2024-10-22

**Authors:** Raghad Alsheikh, Ádám Haimhoffer, Dániel Nemes, Zoltán Ujhelyi, Pálma Fehér, Liza Józsa, Gábor Vasvári, Ágota Pető, Dóra Kósa, Lajos Nagy, László Horváth, Bence Balázs, Ildikó Bácskay

**Affiliations:** 1 Department of Pharmaceutical Technology, Faculty of Pharmacy, University of Debrecen, 4032 Debrecen, Hungary; alsheikh.raghad@pharm.unideb.hu (R.A.); haimhoffer.adam@pharm.unideb.hu (Á.H.); nemes.daniel@pharm.unideb.hu (D.N.); ujhelyi.zoltan@pharm.unideb.hu (Z.U.); feher.palma@pharm.unideb.hu (P.F.); jozsa.liza@pharm.unideb.hu (L.J.); vasvari.gabor@pharm.unideb.hu (G.V.);; 2 Doctorate School of Pharmaceutical Sciences, University of Debrecen, 4032 Debrecen, Hungary; 3 Institute of Healthcare Industry, University of Debrecen, 4032 Debrecen, Hungary; 4 Department of Applied Chemistry, Institute of Chemistry, Faculty of Science and Technology, University of Debrecen, 4032 Debrecen, Hungary; nagy.lajos@science.unideb.hu; 5 Department of Pharmaceutical Surveillance and Economics, Faculty of Pharmacy, University of Debrecen, 4032 Debrecen, Hungary; horvath.laszlo@pharm.unideb.hu; 6 Institute of Medical Microbiology, Faculty of Medicine, University of Debrecen, 4032 Debrecen, Hungary; balazs.bence@med.unideb.hu

**Keywords:** in situ gel, poloxamers, drug delivery systems, thermosensitive gels, ocular drug delivery, antibiotics, Azithromycin, Ofloxacin

## Abstract

In situ gels have been developed as an innovative strategy to prolong corneal residence time and enhance drug absorption compared to traditional eye drops. Our study aimed to formulate an ophthalmic in situ gel with a combination of two thermosensitive poloxamers, P407 and P188, in an optimal ratio not only to increase the time of action but also to increase the solubility of selected antibiotics for the treatment of ophthalmic infections. Two BSC II class substances, Azithromycin and Ofloxacin, with different mechanisms of action, have been incorporated into the in situ gel system after determining their solubility. The antibiotics-loaded in situ gel formulation was evaluated for its clarity, pH, rheological properties, and gel characteristics of gelling time, temperature, and capacity. The formulation demonstrated satisfactory clarity, appropriate pH, effective gelation properties in simulated tear fluid, and suitable rheological characteristics. In addition, APIs release insight has been studied through a dissolution test, and the effectivity against sensitive and resistant bacterial strains has been proved through the antimicrobial study. Therefore, our in situ gel system based on thermosensitive poloxamers, with two hydrophobic antibiotics, AZM and OFX, can be considered a valuable approach for ophthalmic drug delivery with an enhancement of the antibiotics bioavailability through increasing the contact time with the ocular surface and enhancing patient compliance.

## 1. Introduction

The eye is a complex and crucial anatomical organ within the human anatomy, representing a vital organ that has had the attention of researchers for decades [1]. Ocular infections are common in various regions of the world, occurring in different parts of the eye as conjunctivitis, keratitis, and endophthalmitis, posing significant threats to vision, potentially resulting in blindness if left unattended [2,3]. Pathogenic bacteria, including gram-positive and gram-negative bacteria such as *Staphylococcus aureus*, *Staphylococci*, *Streptococcus pneumoniae*, and *Pseudomonas aeruginosa*, are major contributors to various forms of eye-related infections [4]. The damage inflicted by ocular infections without timely and appropriate therapy, poses significant risks to eye health, increasing the likelihood of visual impairments or irreversible vision loss [5].

Indeed, traditional topical ophthalmic dosage forms like solutions and ointments have been widely used for ocular drug delivery. However, they come with certain limitations that can affect their effectiveness. The unique structure and physiology of the eye present several challenges for ocular drug delivery. The cornea, the outermost layer of the eye, is a major obstacle to drug penetration due to its hydrophilic and lipophilic layers. Moreover, the drainage and natural eye defense mechanisms contribute to the challenges of maintaining effective drug concentration on the ocular surface [6], such as rapid pre-corneal drug elimination through tear production. In addition, the short residence time limits the duration of therapeutic action, necessitating the frequent application of eye drops and triggering blurred vision, resulting in patient compliance issues [7].

To overcome these limitations, researchers and pharmaceutical companies have been exploring innovative strategies to improve ocular drug delivery by increasing the corneal contact time [8]. Among all these strategies, the in situ gels are considered a novel, unique, smart, and promising approach for ocular drug delivery over conventional formulations. The major innovation of these gels is related to the unique characteristic of the ‘sol-gel’ transition [9]. In essence, before administration, at room temperature (25 °C), the gels will be in a solution form, and immediately after administration, once contacting the physiological environment of the eye at (35 °C), the system undergoes phase transition leading to the gelation of the product. The in situ forming gels are liquid formulations providing easy, safe, and accurate installation. However, their rapid gelation after the installation exhibits new viscoelastic properties that limit pre-corneal elimination and extend the residence time over the ocular surface [10,11]. This prolonged contact enhances bioavailability and reduces systemic absorption, ultimately leading to enhanced therapeutic efficacy. Studies in both rabbits and humans have shown that aqueous gels improve ocular drug bioavailability and produce better therapeutic responses compared to standard eye drops [12]. Ammar et al. have proved that the formulation of dorzolamide hydrochloride as an in situ gel nanoemulsion has increased drug bioavailability and improved its therapeutic effectiveness compared to standard drug solutions or market products, with a better drug penetration by prolonging its residence time, thus reducing the frequency of applications needed and improving patient compliance [13]. Furthermore, the results of a study by Asasutjarrit et al., have proved the improvement of ophthalmic bioavailability of diclofenac sodium compared to commercial eye drops, as formulating diclofenac sodium in situ gel with the combination of poloxamer 407 and poloxamer 188 can enhance the absorption drug in the eyes and extend its elimination time from the aqueous humor [14]. Moreover, in situ ocular gels reduce the frequency of application, thereby improving patient compliance and quality of life [15]. Currently, several in-situ gel formulations are commercially available for ocular drug delivery. For example, Timoptic-XE^®^, containing timolol maleate (0.25% and 0.5%) in gellan gum, has been on the market since 1994 and is used topically to treat glaucoma [1]. Various therapeutic agents, including antibiotics, beta-blockers, and NSAIDs have also been successfully delivered using in situ gelling systems, which underscores the potential of in situ gelling formulations as a promising future approach for drug delivery [16].

The sol-gel transition can be triggered by biological stimuli and physicochemical changes like temperature, pH, and ion concentrations in lachrymal fluid [17]. This transition achieved by a variety of polymeric compositions, provides advantages such as easy administration close to the conventional eye drops, prolonged retention at the site of action, and sustained drug release through the gel network [18].

Azithromycin (AZM), the first azalide antibiotic derived from erythromycin within the macrolide class [19], gained FDA approval for clinical use in 1992 [20]. Exhibiting broad-spectrum efficacy against gram-positive, gram-negative, and atypical bacteria, including *Hemophilus influenzae*, *Staphylococcus aureus*, and *Streptococcus pneumonia* [21], and functioning by inhibiting bacterial protein synthesis, through reversible binding to the 50S ribosomal subunit of bacteria, which is a crucial component involved in the synthesis of proteins [22]. AZM is known for its long half-life and offers versatility in administration through oral, parenteral, or ophthalmic solutions [23]. Macrolide antibiotics, including AZM, come with limitations such as low solubility and restricted cellular permeation [24]. The physicochemical characterization of AZM and according to the World Health Organization (WHO) [25], AZM is classified as a Class II drug (high permeability/low solubility) in the biopharmaceutical classification system (BCS). Although the high permeability of AZM is an advantageous characteristic, the low solubility may serve as a rate-limiting step in its absorption process, potentially affecting its bioavailability [26]. In specific clinical scenarios, especially in ocular surface infections, topical treatment of antibiotics emerges as a preferred alternative to systemic therapy due to direct treatment at the infection site leading to reduction of the systemic exposure risks while concurrently mitigating bacterial resistance development, a significant advantage for antibiotics in topical administration [27,28].

Ofloxacin (OFX) emerges as a potent second-generation fluoroquinolone antibiotic [29], characterized by its expansive and versatile antibacterial activity against a spectrum of pathogens. In vitro analyses reveal OFX’s superior potency, particularly in combating most gram-negative bacteria, many gram-positive bacteria, and some anaerobes [30]. OFX applications extend to both systemic and localized infections, with the approval for corneal ulcer treatment in 1996. Investigations into the pharmacokinetics of second-generation fluoroquinolones, including OFX, underscore their effective corneal stromal penetration and targeted ocular drug delivery, achieving minimum inhibitory concentrations against ocular pathogens [31]. Pharmacokinetically, OFX exhibits a 6–7 h half-life, and its classification as a BCS II drug indicates low water and methanol solubility coupled with high permeability [32]. Mechanistically, the antibacterial efficacy of OFX stems from its ability to inhibit essential enzymes in bacterial DNA replication, transcription, repair system, and recombination, such as the bacterial DNA gyrase enzyme which is involved in the supercoiling of bacterial DNA, and the topoisomerase IV enzyme which plays a role in the separation of replicated DNA strands during cell division. By interfering with these enzymes, OFX disrupts the normal bacterial DNA processes, leading to the inhibition of bacterial cell division and ultimately causing bacterial cell death [33].

Poloxamers, also known as Pluronics, are a class of non-ionic surfactants that belong to the family of block copolymers. They are amphiphilic, meaning they have both hydrophilic and hydrophobic components of polyethylene oxide (PEO) and polypropylene oxide (PPO) units [34]. Indeed, natural polymers have garnered significant application in various industries due to their advantageous properties. In pharmaceuticals, they stand out for their biocompatibility, leading to fewer adverse reactions, and their natural degradation into harmless compounds [35].

Among these, poloxamer 407 (P407, 73 wt% PEO, 12,600 g/mol) and poloxamer 188 (P188, 80 wt% PEO, 8400 g/mol) have emerged as prominent players in drug delivery research, particularly in the development of temperature-sensitive hydrogels. Moreover, because of their good water solubility, low biotoxicity, strong biocompatibility, and ability to control drug release over time, these thermosensitive gels find extensive use in the biomedical field [36,37]. In addition, their unique composition of hydrophilic PEO and hydrophobic PPO blocks, allows them to self-assemble into micelles after reaching a critical micelle concentration (CMC) and/or a critical micellization temperature (CMT). At lower temperatures, the poloxamers solution remains in a liquid form, due to the hydrogen bonds between the polymer blocks and water molecules. However, when the concentration of poloxamers increases above the CMC, or the temperature rises above the CMT, dehydration of the PPO blocks occurs and forms a hydrophobic core inside, while the outer PEO blocks remain hydrated and form a hydrophilic shell [38,39,40]. Polymeric micelles exhibit a distinctive core-shell architecture, wherein the hydrophobic core serves as a space for drug loading, providing a conducive environment for the encapsulation of hydrophobic drugs [41]. This amphiphilic core-shell structure enables polymeric micelles to maintain stability in biological fluids while effectively encapsulating water-insoluble active pharmaceutical ingredients (APIs) within their core. As a result, the solubility and bioavailability of such drugs are significantly enhanced [42], as well as facilitating their absorption across biological barriers, leading to more efficient delivery and better therapeutic outcomes [43]. The histopathology studies in Barse et al.’s research demonstrated that the use of P407 in the in situ gel formulation with the drug dorzolamide hydrochloride was safe for ocular use. Goat corneas treated with the formulation showed normal ocular surface structures and maintained normal cell morphology, with no signs of hemorrhage or necrosis. The results confirm that the P407-based formulation is non-irritating to the eye, making it a safe alternative to conventional eye drops [44]. Moreover, a study conducted by M.A. Fathalla et al. focused on developing and evaluating in situ gel formulations using poloxamer 407 and poloxamer 188 for sustained ocular delivery of ketorolac tromethamine (KT). The safety of these formulations was confirmed by the hen’s egg test on the chorioallantoic membrane (HET-CAM) and bovine corneal opacity and permeability (BCOP) tests, which demonstrated non-irritancy and protective effects against irritants. Additionally, MTT cytotoxicity assays showed acceptable cell viability for the KT-loaded gels compared to control samples, confirming that the formulations were safe for ocular use. These findings suggest that using P407 and P188 KT-loaded in situ gels offers a promising, non-irritating option for ocular drug delivery [7].

The development of an optimized in situ gel using two poor water-soluble antibiotics with a combination of two temperature-sensitive poloxamers, P188 and P407 as thermos-responsive gelling agents, is our strategy employed to create an expanded antibacterial spectrum with sustained drug release for enhanced ocular bioavailability, thereby potentially improving the efficacy and convenience of topical dosing antibacterial therapy in ophthalmic applications.

## 2. Materials and Methods

### 2.1. Material

P407 (Kolliphor^®^ P407) and P188 (Kolliphor^®^ P188) were purchased from Merck (Merck Kft., Budapest, Hungary). Azithromycin dihydrate (AZM; >98%) and Ofloxacin (OFX; >98%) were bought from TCI (Zwijndrecht, Belgium).

All materials were stored under dry, cool, and dark conditions until the measurements and sample preparation. In addition, all solvents used were of adequate analytical quality. Distilled water was prepared in the laboratory using a water purification system.

### 2.2. Methods

#### 2.2.1. Design of Experiment

The ideal poloxamers ratio was optimized using a full-box experimental design. The independent variables were the amount of P407 and the amount of P188, which were considered critical parameters of the eye drops with an effect on in situ gelling. These two experimental factors were varied in the design, at three levels in nine runs overall. The amount of P407 was set to either 5, 15, or 20 g per 100 g of purified water while the amount of P188 was either 1, 3, or 5 g per 100 g of purified water. This design was employed to investigate the quadratic response surface and to construct a second-order polynomial model using TIBCO Statistica^®^ version 13.4 (Statsoft Hungary, Budapest, Hungary). The 3D response surface plots for viscosity and pH were plotted according to the regression model by keeping one variable at the center level. The relationship of the variables in the response can be analyzed by the following second-order equation:

Y = β_0_ + β_1_ × X_1_ + β_2_ × X_2_ +…+ β_p_ × X_p_ (1)

where Y is the response variable; β_0_ is a constant; β_1_, β_2_, and β_p_ are linear coefficients; X_1–p_ are the main effect factors.

The formulation and characterization of the in situ gels process was carried out using a “cold method”. Initially, sufficient quantities of P407 and P188, as mentioned in Table 1, were gradually added into 100 g of water, within a beaker, with continuous stirring using a magnetic stirrer set at 300 rpm, overnight, at room temperature (25 °C) [45].

The prepared in situ gel formulations were visually inspected for clarity, color uniformity, and the presence of particles. This examination was conducted under suitable light against both white and dark backgrounds to ensure a thorough assessment [46].

The pH of the gels was measured using a calibrated pH meter (Mettler Toledo FiveEasy pH meter, Mettler-Toledo Kft., Budapest, Hungary). The measurements were conducted in triplicates with three, individually prepared samples [47].

Viscosity measurements were carried out with the Zetasizer Nano ZSP device, updated with a microrheology module (Malvern Instruments Ltd., Malvern, UK). In the field of micro-rheology, micron-sized spherical tracer particles are incorporated into the sample to monitor their motion, utilizing dynamic light scattering (DLS) techniques employing a coherent monochromatic light source and detection optics to analyze intensity fluctuations in scattered light. According to the instructions of the manufacturer, first, we evaluated tracer particle-sample matrix interactions through zeta potential tests in the dispersant alone (5 µL neat tracer 655 nm melamine particle suspension to 10 mL water), and then in the presence of the sample by adding one drop of the dispersed (diluted) sample to approximately 5 mL of the previously prepared tracer dispersion with sufficient mixing. After no interaction was found, we employed another pre-measurement step to estimate the tracer concentration needed to achieve a relative intensity ratio of 95%. In our case, 5 μL tracer suspension was added to 200 μL of our poloxamers samples [48]. Once compatibility between our poloxamers samples and the tracer has been confirmed and the appropriate tracer concentration has been determined, viscosity measurements of our nine samples were conducted at temperatures of 25 °C and 35 °C in triplicates.

#### 2.2.2. Phase Solubility

In order to obtain the calibration curve, a stock solution of AZM with Acetonitrile was prepared. Serial dilutions to six different concentrations within the range of 0.5, 0.3, 0.1, 0.05, 0.03, and 0.01 mg/mL were prepared from the stock solution.

After having the calibration curve, a sufficient amount of AZM (10 mg) was added to serial dilutions of the poloxamers gel mixture (20 g of P407 and 5 g of P188 in 100 g of purified water) in a concentration of 4, 6, 8, 10, 12, 14, and 28%, prepared at room temperature (25 °C), mixed for 24 h, centrifuged with 5000 rpm for 20 min with (25 °C), and finally filtered through 0.45 µm pore size filters.

Samples were introduced into the LC/MS chromatographic system (in SIM mode) consisting of a Waters 2695 Separations Module with a thermostable autosampler (5 °C) and a column module (35 °C), a VDSphere PUR 100 C18-M-SE reverse phase C-18 column (4.6 × 150 mm, 5 μm) (VDS Optilab Chromatographie Technik GmbH, Berlin, Germany) coupled with a MicroTOF-Q type Qq-TOF MS instrument (Bruker Daltonik, Bremen, Germany) using an electrospray ion source (ESI) with positive ion mode. The flow rate and the injected volume were 1.0 mL/min and 10 mL, respectively, in all cases. A splitter was applied between the HPLC and MS obtaining a flow rate of 0.1 mL/min for the ESI ion source. The mass spectra were calibrated externally using the exact masses of the clusters generated from the electrosprayed solution of sodium trifluoroacetate (NaTFA). The recorded mass spectra were evaluated with the DataAnalysis 3.4 software from Bruker.

#### 2.2.3. Preparation of the Loaded Gel

A previously determined amount of AZM was added to the unloaded gel which contained 5 and 20 g of P188 and P407 in 100 g water, respectively. In order to determine the OFX solubility, we employed a different method. Specifically, we incrementally added 1 mg portions of OFX to the unloaded gel, followed by centrifugation at 5000 rpm for 20 min at 25 °C to check whether it had fully dissolved. After confirming its solubility in the poloxamer mixture, we applied the same method to the in situ gel loaded with AZM, by mixing different concentrations of OFX with the AZM-loaded gel, and stirring continuously overnight until it dissolved. All formulations underwent clarity testing through visual inspection of the samples to detect any transparent or colored particles. The pH values of the different gels were measured using a calibrated pH meter. We found that the maximum amount of OFX that could be dissolved in the gels without precipitation was 1.25 mg/g. The formed in situ gels were considered as the final formulation and were stored at room temperature until further use.

#### 2.2.4. Determination of Gelation Time and Temperature

The gelation characteristics of our final formulation were evaluated through two experiments employing the tube inversion method. In the determination of gelation time, 2 mL of the gels were placed in glass tubes and incubated at room temperature (25 °C) before being transferred to a water bath set at (35 °C ± 1), following the protocol outlined by other researchers’ studies [49,50,51,52]. The tube was inverted at regular intervals (every 10 s) until the gel no longer flowed, indicating gelation completion. Similarly, for determining gelation temperature, as described by Wei et al., 2 mL of the sample solution was initially placed in a transparent glass test tube and immersed in a water bath set at (25 °C). The temperature was gradually increased at a rate of (1 °C) every 2 min, and the gelation temperature was recorded when the formulation ceased to move upon tilting the tube [53].

#### 2.2.5. Gelling Capacity

The examination of the gelling capacity was conducted by firstly staining the loaded in situ gel sample using Patent Blue V (E 131) at a final concentration of 0.01% (*w*/*v*) to impart a blue color, and then slowly placing a drop of the formulation into a glass test tube containing 2 mL of freshly prepared simulated tear fluid (STF) with a pH of 7.4 equilibrated at (35 °C). Evaluation of the gelling capacity of the formulation was based on visually observing the time taken for its gelling formation and its duration of retention [54,55]. STF was prepared with the composition of 0.67% sodium chloride, 0.20% sodium bicarbonate, 0.008% calcium chloride dihydrate, and distilled water to 100 mL [56].

#### 2.2.6. Fourier–Transform Infrared Spectroscopy (FT-IR)

FT-IR analysis was carried out to assess the physicochemical interaction of APIs, as well as the APIs with poloxamers. The FT-IR spectra of a pure AZM powder, pure OFX powder, unloaded gel, 1.125 mg/g AZM dissolved in the final poloxamers gel, 1.25 mg/g OFX dissolved in the final poloxamers gel, and two APIs loaded gel were recorded by using a JASCO FT-IR 4600 type (ABL&E-JASCO, Budapest, Hungary) apparatus coupled with a Zn/Se ATR PRO ONE Single-Reflection ATR accessory. Each sample was directly placed on the cleaned crystal of the equipment. Spectral scanning was conducted 24 times in the range between 4000 and 500 cm^−1^ at a resolution of 1 cm^−1^ to obtain a smooth spectrum. Corrections of environmental CO_2_ and H_2_O used the built-in method of the software [57].

#### 2.2.7. Osmolality Measurement

The total osmolality of the unloaded and loaded gel formulations was determined using the OSMOMAT 070 vapor pressure osmometer (Gonotec GmbH, Berlin, Germany). Sampling was conducted for 4 min at a measurement temperature of (25 °C). Ultrapure (Type 1) water from a Millipore Direct-Q 5 UV system (Millipore SAS, Molsheim, France) was selected as the solvent. Before each experiment, the baseline was established with ultrapure water and the device was calibrated with 0.9% *w*/*v* sodium chloride solution. Following calibration with the calculated cell constant, measurements were taken for both loaded and unloaded gel samples. Each liquid was applied to the sensors twice, with the second drop utilized for measurement purposes. The resulting osmolality of the samples was expressed in mOsmol/kg, and the experiments were carried out four times [58].

#### 2.2.8. Rheological Behavior of the Loaded In Situ Gel

The apparent viscosity of the loaded in situ gel sample was assessed using a Rheolab QC rheometer (Anton Paar Hungary Ltd., Budapest, Hungary), which was equipped with a concentric cylinder-jacketed measuring cell. This cell was linked to a Viscoterm VT 2 water bath. Viscosity data were captured using RheoPlus software version 3.1. Measurements were taken across temperatures ranging from (25 °C) to (39 °C), at intervals of (2 °C), using a shear rate ranging from 0.01 to 100 1/s at 9 points [59]. At each measurement point, shear stress, speed, torque, and apparent viscosity were measured and calculated respectively. The formulation was tested in three, individual experiments with a 25 mL sample each time.

#### 2.2.9. Dissolution Test

**Figure 1:**
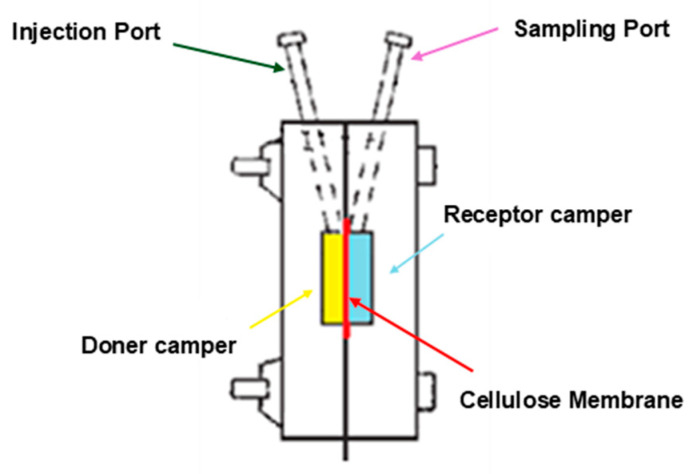
Diagram of the dissolution apparatus; The donor chamber (yellow box) contains the loaded in situ gel formulation and the receptor chamber (blue box) contains the dissolution medium (phosphate buffer, pH 7.4). The 50 kDa cellulose membrane (red line) is positioned between the two chambers through which the drug can diffuse. The injection port is where our formulation is introduced into the system for testing, and the sampling port is used to take samples from the receptor chamber at specific time intervals (15 min, 30 min, 1 h, 2 h).

The dissolution test of our loaded gel was performed using IN-LINE EQUILIBRIUM CELL apparatus (Bel-Art Products, Wayne, NJ, USA), with 4 parallel in-line cavities with dimensions of 152 mm L × 25 mm W × 76 mm H, and containing 1 mL in every half-cell. The dissolution medium used in this experiment was phosphate buffer (pH 7.4) and a 50 kDa cellulose membrane was utilized to separate the two chambers (Figure 1). The dissolution medium was removed at different time intervals (15 min, 30 min, 1 h, 2 h) and analyzed for AZM and OFX. The amount of the two antibiotics, AZM and OFX, released during a sampling period was measured using liquid chromatographic/mass spectrometry (LC/MS) as mentioned in the phase solubility method [60].

#### 2.2.10. Microbiology Study

In killing studies, the following *S. aureus* clinical samples of the Institute of Medical Microbiology were used: 13746 and 13188 are OFX and AZM sensitive, 13499 are OFX sensitive and AZM resistant, and 12446 are resistant for both OFX and AZM. The time-kill method was carried out at two concentrations, testing the original formulation and its diluted form as well. The non-diluted test tubes contained 100 µL of 1 × 10^6^ cells/mL cell suspension and 1000 µL of the loaded formulation. The diluted tubes contained 300 µL of 1 × 10^6^ cells/mL cell suspension and 300 µL of the loaded formulations with 2400 µL of the previously mentioned STF. Control experiments were carried out with unloaded gel formulation and without any formulation [61]. The test tubes were incubated for 8 h at (35 °C) and aliquots of 100 µL were removed after 0, 2, 4, 6, 8, and 24 h of incubation. The tenfold serial dilutions were prepared, and samples of dilutions (4 × 30 µL) were plated onto a single Mueller–Hinton plate and incubated at (35 °C) for 48 h. Tests were carried out in duplicates and mean values were presented. In any given concentration, where results differed from each other by more than 5%, a third experiment was carried out [62].

## 3. Results

### 3.1. Gel Characteristics: Design of Experiment Studies on Visual Appearance, Homogeneity, Clarity, Determination of pH, and Viscosity

Clarity and absence of particles in the ocular formulations are highly desirable characteristics for the ophthalmic application. Visual examination of our nine gels formulated with varying ratios of P188 and P407 as shown in Table 1, exhibited transparency and colorlessness, crucial for maintaining clear vision and patient acceptance. Notably, all preparations, as confirmed by visual inspection, were devoid of any particles, ensuring smoothness upon application.

**Figure 2:**
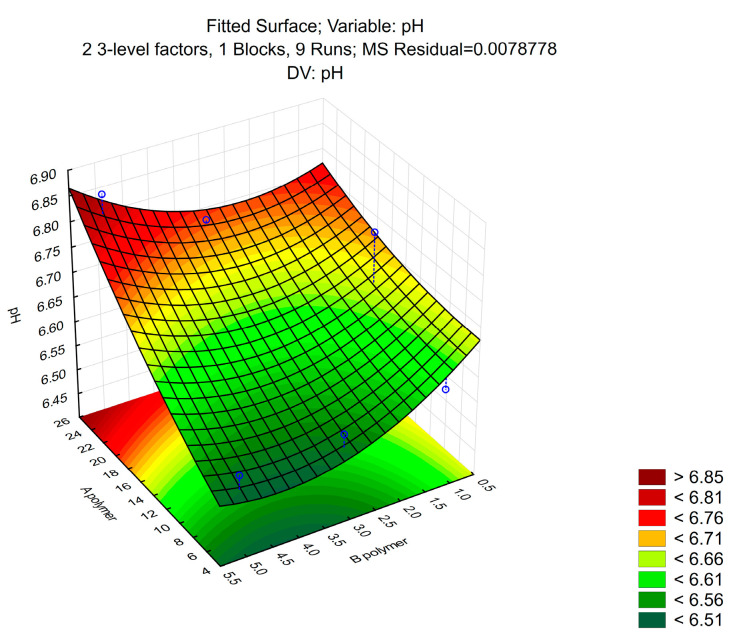
The pH values of the different ratios of P407 and P188 gels. (n = 3).

The pH values of ophthalmic formulations are strictly controlled, as physiological damage can occur when applying too acidic or too alkalic preparations. As seen in Figure 2, the pH value of all samples ranged between 6.65 ± 0.03, which ensures minimal irritation during ocular administration, and fitting the normal human tear fluid pH.

**Figure 3:**
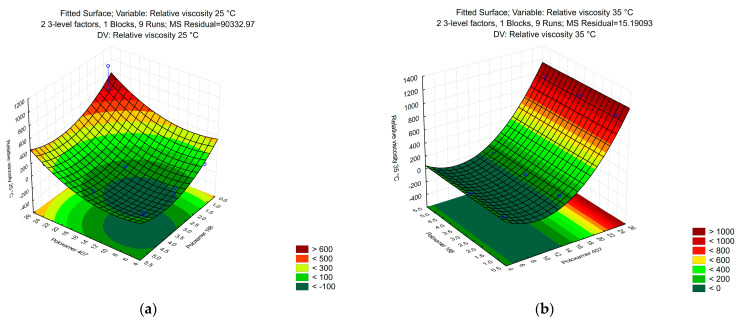
The relative viscosity of the different ratios of P407 and P188 gels measured by DLS microrheology at 25 °C (**a**) and at 35 °C (**b**). (n = 3).

Poloxamer mixtures are thermo-reversible, meaning that their viscosity changes with the temperature. We aimed to create an ophthalmic formulation, which is easy to drop from a container at room temperature but has a long residence time on the corneal surface. As seen in (Figure 3b), only those formulations had high viscosity, which had a high P407 concentration. However, certain ratios at (25 °C) already had high viscosity, which prevented dropping (Figure 3a). For further study, we chose the ratio of 5 g of P188 and 20 g of P407 that had favorable rheological properties at both temperatures. A higher concentration of P407, up to 25 g per 100 g of purified water, was found to start forming a gel at room temperature, thus, we limited its amount to 20 g in order to have the necessary gel strength for sustained drug release and low viscosity at room temperature. The P188, as a temperature modifying agent, had to be added in order to decrease the concentration of P407 as well as ensure the appropriate fluidity and help enhance the solubility of the drug without compromising the gel structure.

### 3.2. Phase Solubility Profile

**Figure 4:**
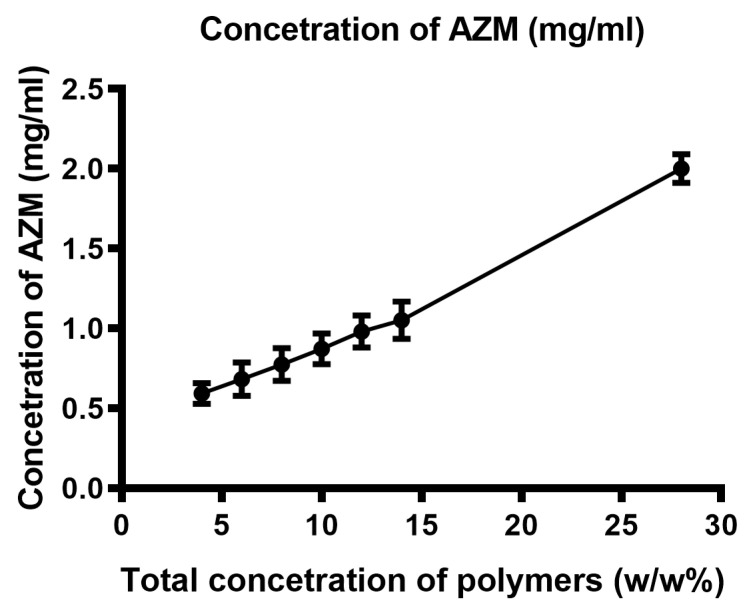
Phase solubility study of AZM in the presence of a merged concentration of the two poloxamers (20 g of P407 and 5 g of 5 g of P188 in 100 g of purified water).

Through a phase solubility study conducted using LC/MS to assess the solubility of AZM in the poloxamers gel, we could demonstrate the solubilization effect of poloxamers on AZM. We have found that the solubility of AZM in the poloxamers matrix was 1.125 mg/g. We have observed that poloxamer micelles possess the capability to enhance the solubility of AZM. Results showed a direct correlation between the concentration of poloxamers and the solubility enhancement of AZM, which means that increasing the concentration of poloxamers led to a proportional increase in the solubility of AZM (Figure 4).

The amphiphilic nature of poloxamer copolymers facilitates the formation of micellar structures, where hydrophobic PPO blocks aggregate in the core while hydrophilic PEO blocks form the outer shell. This unique architecture enables the encapsulation of hydrophobic molecules, including insoluble APIs like AZM, within the core of the micelle, thereby further enhancing its solubility in aqueous media.

### 3.3. Preparation of Loaded Gel

Aqueous solutions of P188 and P407 were prepared by dispersing them in distilled water with constant stirring at (25 °C) for 24 h to make a clear solution. The next day, the optimum concentration of AZM was added to the gel and kept for dissolving overnight with continuous stirring using a magnetic stirrer. Finally, OFX was added to the gel and mixed well. The composition of the gel, including the exact amounts of active ingredients and excipients, is shown in Table 2.

Visual testing of our final in situ gel formula proved no visible foreign matter, with a slightly yellowish color occurring due to the homogeneously distributed OFX. The pH of our loaded in situ gel formulation was (7.4), which ensured the comfort, safety, and efficacy of the preparation.

### 3.4. Gelation Time and Temperature Findings

Thermo-sensitive in situ gelling systems exhibit a sol-gel transition close to physiological temperatures due to significant changes in polymer-water interactions, mediated by hydrophobic PPO and hydrophilic PEO groups within the polymer structure. The gelation temperature for our loaded in situ gel formulation was determined to be approximately (31 °C), with gelation occurring within 30 s, followed by incubation of the sample at (35 °C) water bath through a tube inversion technique.

### 3.5. Gel-Forming Capacity

Testing the gelling capacity of the in situ gel formulations is necessary to determine the time needed for the sol-gel transition and the time needed for the formed gel to be dissolved. The visual observation of the gelling formation of our loaded gel was 30 s and the dissolving time needed was 5 min.

### 3.6. FT-IR Spectal Analysis

**Figure 5:**
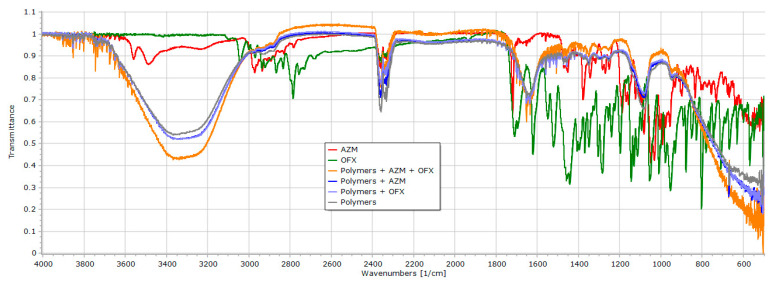
FT-IR spectra of pure AZM powder, pure OFX powder, unloaded gel, OFX dissolved in poloxamer gel, AZM dissolved in poloxamer gel, and the loaded gel.

The FT-IR spectroscopy measurement technique was carried out to determine the APIs-gel polymers’ compatibility and their possible interactions. The IR spectra of pure AZM and OFX powders, poloxamer gel formulation, and each API-poloxamers gel were taken separately and later compared with the spectra of the final loaded gel as shown in Figure 5.

The detailed comparison of the spectra of the AZM and OFX powders and the respective loaded gel formulation reveals that there are no observable shifts of the peaks. As the spectra are nearly identical and they lack any significant changes, it is highly possible, that the secondary chemical bonds between the drugs and the poloxamers are weak, and they cause no stability issue in the case of any of the active substances.

### 3.7. Osmolality Determination

The osmolality of ophthalmic preparations is an important factor, given that a too high deviation from the physiological tear fluid can result in pain during administration. The osmolality of unloaded and loaded gels was measured with a vapor pressure osmometer. We found that the loaded gel had slightly lower osmolality than the unloaded gel (Table 3), and both values can be considered hypoosmotic, which needs further adjustment through the addition of different tolerable salts like sodium chloride. The exact amount will be determined after the necessary stability studies are carried out.

### 3.8. Viscosity Rheological Properties

**Figure 6:**
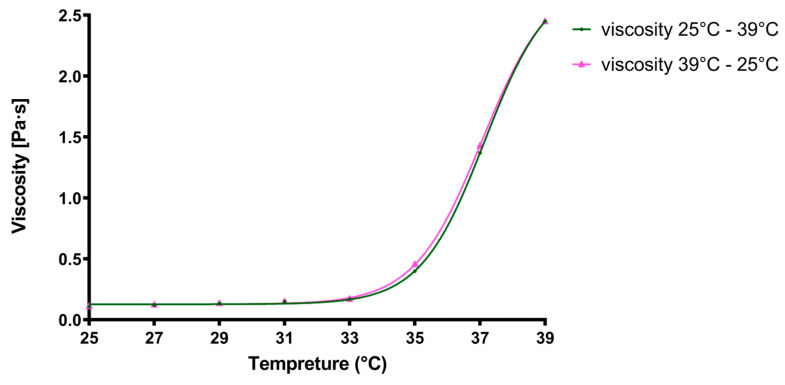
Apparent viscosity of the final formulation in case of increasing (green line) or decreasing (pink line) temperature. Each plotted value represents the final viscosity at 100 1/s shear rate after increasing the shear rate from 0.01 to 100 1/s at 9 points. (n = 3).

Application at room temperature enables the in situ gels to be droppable, while it is required that at the ocular surface, where the temperature ranges between (33 °C to 37 °C) [63,64], gelation must be complete, increasing viscosity and thus extending the retention time of the formulation, which prolongs the action of APIs. In order to investigate the influence of temperature on the rheological properties of the loaded gel formulation, the samples were first heated up from (25 °C) to (39 °C) and then cooled down to the initial temperature, and at each measurement point the viscosity was measured with increasing shear rate. As can be seen in Figure 6, the viscosity of the formulation did not differ during cooling down when compared to the heating up which proves the stable rheological nature of the gel.

**Figure 7:**
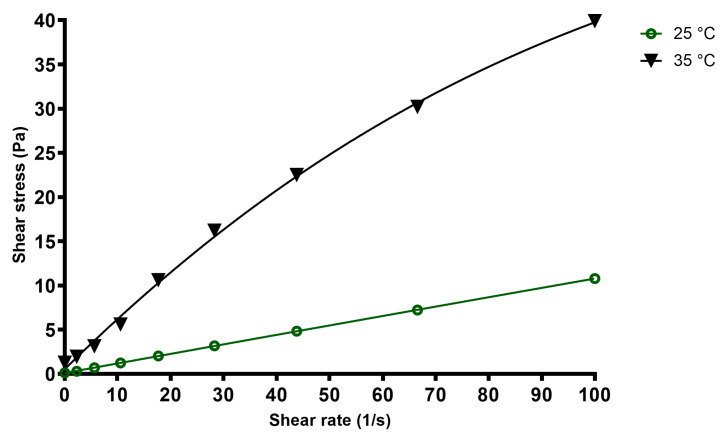
Relative viscosity of the final formulation in case of increasing (green line) or decreasing (black line) temperature. Each plotted value represents the final viscosity at 100 1/s shear rate after increasing the shear rate from 0.01 1/s at 25 steps. (n = 3).

Flow curves of the final formulation at different temperatures can be seen in Figure 7. At room temperature, while dropping, the in situ gel acts as a Newtonian fluid, while on the ocular surface, at (35 °C), pseudoplastic behavior can be observed, as the apparent viscosity decreases with increasing shear stress. This is advantageous for the formulation as during blinking or in case of rapid eye movements, shear stress increases, and lower viscosity increases the spreading of the formulation on the ocular surface.

### 3.9. Dissolution Profile Analysis

**Figure 8:**
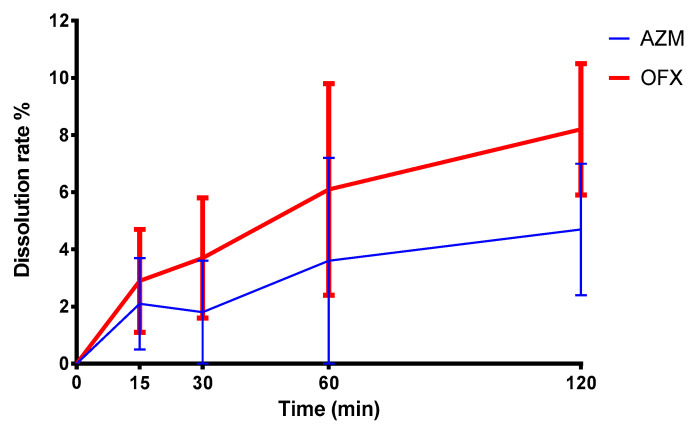
Dissolution test of the formulation, utilizing a dialysis cell with cellulose membrane. The means of three parallel experiments and their ±SEM are plotted.

In order to study and verify the liberation of the drugs from the formulation, a dissolution test was carried out in a dialysis cell. As can be seen in Figure 8, without external agitation, like blinking or eye movements, the liberation of the two antibiotics is slow from the polymer matrix, which provides the elongated time of action. OFX due to its higher water solubility is more rapidly dissolves into the external media.

### 3.10. Microbiological Results 

**Figure 9:**
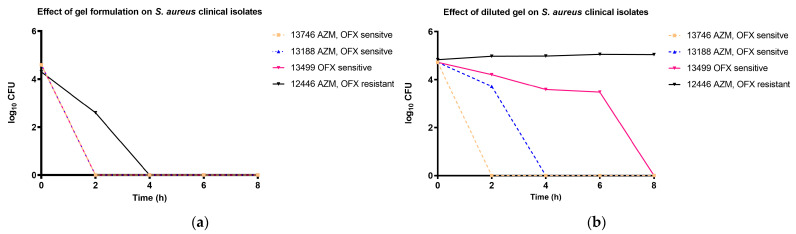
Time-kill tests of the final formulation in different concentrations against clinical strains of *S. aureus*. (**a**): The non-diluted test was carried out by mixing 100 µL of cell suspension and 1000 µL of the gel. (**b**): The diluted test was carried out by adding 300 µL of the gel into 2700 µL of STF and cell suspension.

The effectiveness of the formulations was tested in vitro against *S. aureus* in time-kill experiments. Instead of reference strains, we chose the clinical isolates of the University of Debrecen, isolated from patients to create a more relevant test environment. Through previous testing, two strains that were sensitive to both antibiotics, one strain that was OFX sensitive, and one strain that was double resistant for both antibiotics were chosen to prove the bioavailability of both OFX and AZM against bacterial cells. The formulation was tested in its original concentration diluted only with the cell suspension (10:1 ratio) and in a ten-fold dilution (1:10 ratio) to simulation dilution in the tear fluid after application. As seen in Figure 9a, the non-diluted formulation had such a high concentration of antibiotics, that after 4 h, the resistance of the 12,446 clinical isolates was broken and all bacterial cells were terminated. In the case of the diluted gel, presented in Figure 9b, the 12,446 clinical isolates had only limited growth, and the AZM-resistant 13,499 clinical isolates were terminated after 8 h of incubation after continuous decline in cell number, due to the increasing concentration of OFX.

## 4. Discussion

Traditional eye drops are one of the most frequently used methods for delivering ophthalmic drugs, as the liquid solutions are easy and convenient to administer to patients. However, their rapid drainage from the eye results in a shortage of their ocular residence time which leads to a decrease in effective drug concentration.

To improve the bioavailability of a drug, novel delivery approaches of prolonged-release formulations have gained attention and have been developed in recent years. The in situ gel system is one of the innovative approaches in ophthalmic drug delivery aimed at improving the effectiveness of treatments for ocular diseases. They are liquid formulations that transform into a mucoadhesive gel after contact with the ocular surface, triggered by stimuli such as pH alteration, ionic cross-linkage, or temperature changes. This gelation process prolongs the ocular retention time of the drug, consequently leading to enhancement of the drug’s bioavailability and improvement in the therapeutic outcomes while minimizing the need for frequent and increased patient compliance [65].

The thermosensitive in situ gel systems are formulated by thermosensitive polymers, which are non-ionic surfactants consisting of a triblock copolymer of a hydrophobic PPO and hydrophilic PEO and are known for their ability to remain in a liquid state below the low critical solution temperature (LCST), which makes them easy to administer as eye drops, and forming a gel after being exposed to temperatures at or above the LCST [66].

Poloxamers, particularly P188 and P407, are among the few U.S. FDA-approved classes of these nonionic copolymers, known for their biocompatibility and amphiphilic properties. They have been widely studied for their applications in drug delivery, tissue regeneration, and biosurfactants. Poloxamer 407, in particular, has gained FDA approval for use as an inactive ingredient in various pharmaceutical formulations, including ophthalmic, oral, topical, and intratympanic routes. Its thermos-reversible properties make it attractive for formulations like hydrogels, enabling sustained and prolonged drug release. For ophthalmic use, the FDA has approved poloxamer-based formulations, such as gels, solutions, and suspensions [67,68,69]. Previous studies have found that P407 at a concentration of 18% or higher transforms from a liquid to a gel at room temperature. However, the dilution by tears will result in the loss of its gel-forming ability. Thus, 25% P407 is recommended to ensure the proper gelation [70,71]. Nevertheless, ophthalmic formulations with a high poloxamer concentration greater than 20% can cause eye irritation. Therefore, a combination of various in situ gelling polymers is advisable. A mixture of P407 with P188, as a gelation temperature-modifying substance, could enhance the rheological behavior of the in situ gel delivery system by lowering the P407 concentration and reducing eye irritation [72]. In addition, increasing the P407 concentration will decrease the sol-gel transition temperature (T_sol-gel_) to close to room temperature, while increasing P188 raises the T_sol-gel_. This is because P188, being more hydrophilic, disrupts the water layers around P407 molecules, requiring higher temperatures for gelation. However, the P188 concentration cannot exceed 15%, as higher amounts would make the formulation too thick to easily apply to the eye [14].

At the beginning of our research, we aimed to identify the optimal concentration ratio of P407 and P188. Various combinations of the two poloxamers as shown in Table 1 were prepared and evaluated. Among these, the ratio of 20 g of P407 and 5 g of P188 was selected. As shown in Figure 3a, increasing P407 concentration to 25 g resulted in gelation at room temperature (25 °C), which is unsuitable for an easy-to-administer in situ gel delivery system advantages, which has driven us to add 5 g of P188 to reduce the high P407 concentration to get an acceptable viscosity as a liquid solution at room temperature and a high viscous gel at the ocular surface temperature, thereby improving the gelling properties of the formulated in situ gel system. These findings align with a previous study by Huang et al., who demonstrated the effectiveness of a 20.5% P407 and 5.0% P188 combination of the thermosensitive nanoparticulate gelling systems for chloramphenicol delivery [73].

Clarity is a crucial parameter for ophthalmic formulations to avoid blurring vision; our samples of the poloxamer-containing gels and the loaded in situ gel were visually observed to be clear, colorless, and transparent in both liquid and gel states [74]. The pH of the P407 and P188 formulations and the loaded in situ gel formula were measured using a pre-calibrated pH meter and were (6.65 ± 0.03) and (7.4), respectively [75].

Using the LC/MS analytical method, we determined the solubility of AZM to be 1.125 mg/g within the hydrophobic micellar core of the poloxamer matrix of the final formulation. The study of Adeli et al. aimed to enhance AZM solubility and dissolution rate by creating solid dispersions with various concentrations of hydrophilic carrier, Polyethylene Glycols. The findings indicated that both physical mixtures and solid dispersions exhibited higher dissolution rates compared to the drug alone, with the highest solubility of AZM being 253 ± 0.95 μg/mL [76]. Another investigation demonstrated that the solubility of AZM and dissolution rate were enhanced through solid dispersion with urea, as the drug release profile showed higher dissolution rates for both the physical mixture and the solid dispersion compared to the intact drug with AZM solubility of 169.29 ± 2.02 μg/mL [77]. A different study used Mannitol and β-Cyclodextrin as carriers to improve AZM solubility and showed that the solubility of AZM:Mannitol is 7.8 μg/mL, while the solubility of AZM:β-Cyclodextrin is 9.52 μg/mL [78]. Compared to these results, our formulation utilizing poloxamers could reach higher solubility of AZM in water.

Furthermore, the solubility of OFX was measured by adding various amounts of OFX to a fixed mixture of AZM-loaded in situ gel, with continuous stirring for 24 h. OFX being slightly soluble in water [79], was found to have a maximum solubility of 1.25 mg/g. Okonogi et al. showed that the solid dispersions of various aqueous concentrations of urea or mannitol increased the OFX solubility up to 5 mg/mL [80]. Moreover, according to Patil et al.’s research study, the solubility of OFX varies significantly with pH changes. It is higher in acidic conditions with a range of 29–41 mg/mL and lower in neutral and alkaline environments to approximately 3 mg/mL [81]. These results indicate the remarkable solubility-increasing effects of poloxamers, as in the case of AZM, we could surpass the previous literature findings. However, as expected, the solubilizing capacity of the gel matrix is lowered after being loaded with AZM. This is the reason OFX solubility was lower than previous experimental results.

Gelation temperature is the temperature at which the liquid phase transitions into a gel. Based on the previous studies, the effective in situ gel should transform from the low viscose solution at room temperature (25 °C) into a high viscous gel after contacting with the ocular surface, ideally with a temperature range between (28–34 °C), within 5 min [82]. Our findings demonstrate a transformation temperature of (31 °C), with a gelling time of 30 s.

The gel capacity of our antibiotics-loaded in situ gel is defined by its ability to form and maintain a gel state for 30 s to 5 min before dissolving. Ensuring that the gel forms quickly upon contact with ocular tissue is crucial to prevent discomfort and avoid prolonged contact that could lead to irritation, thus enhancing patient comfort and compliance [83].

The FT-IR spectrums for the AZM and OFX powders separately, as well as the loaded and unloaded gels, have indicated the compatibility of the APIs with the poloxamers that have been used in the formulations. The FT-IR spectrum shown in Figure 5 does not exhibit any shifts in the peaks of either the APIs or the poloxamers, suggesting no strong secondary chemical bonds between the APIs and the poloxamers. Our results are consistent with the findings of Maddukuri et al., for the compatibility study of AZM combined with P188 and P407, with no shift or disappearance of the peaks through the FT-IR spectra analyses [84].

The pseudoplastic behavior is a desired property for the ophthalmic in situ gels, ensuring a less viscous gel under the ocular negative reflexes with the shear stress increasing and returning into a more viscous state at rest, helping to prolong the residence time of the drug on the eye surface [85,86]. Both gel formation and thus viscosity increase and pseudoplastic rheology were verified by our measurement as can be seen in Figure 6 and Figure 7. Also, the viscosity values were stable at any temperature and they were not influenced by either ascending or descending temperature of the samples, nor by the elapsed time.

Combining different antibiotics with diverse mechanisms of action in a single formula is a strategy to improve the effectiveness of therapy against resistant bacterial strains, extend the broader spectrum of bacterial targets, reduce dosage requirements, and delay the development of bacterial resistance. For instance, combining aminoglycoside antibiotics with other antibiotic drugs has enhanced the antibacterial effects and reduced the dosage, thus minimizing potential side effects [87]. Moreover, the combination of two antibiotics will overcome the antibiotic resistance limitation. As Omoya and Ajayi’s research has proven, the effect of the combination of different antibiotics on some multiple antibiotic-resistant bacteria prevents resistance to a single antibiotic and reduces the dose-related toxicity [88]. Our in situ gel formulation was designed to incorporate two hydrophobic antibiotics, AZM and OFX. This combination ensures a synergistic effect and offers extensive coverage against a broad spectrum of ocular pathogens with both sensitive and resistant strains, which will enhance the treatment of ocular infections. The formulation was tested against clinical samples isolated from patients of the University of Debrecen. Figure 9a shows that the original concentration of the formulation can have a bactericidal effect even against multi-resistant strains. However, during the application, it can be suspected that because of the higher volume of tear fluid, in vivo results would be closer to the findings of Figure 9b when only the growth inhibition of multi-resistant strains can be reached. It must be noted that the occurrence of such multi-resistant strains is low and it is more likely that the most common strains will be sensitive to either AZM or OFX.

Overall, through the use of two commonly applied poloxamers, the solubility of AZM and OFX was increased in order to create an in situ ophthalmic gel formulation. The exact amount of the poloxamers and the two antibiotics were determined experimentally, outlined by a full-box experimental design. While the increase in water solubility was prioritized, gel formation behavior was also considered, and the final formula was assessed and determined by these two factors. Characterization of the final product verified the suitability of the in situ gel and in vitro antimicrobial experiments proved the effectiveness as well. However, the current findings must be followed by further stability studies in order to confirm the long-term compatibility of the components and the lack the chemical degradation. Moreover, it is needed to properly assess corneal penetration and retention time on in vitro eye models and on in vivo animal models as clinical efficacy in cases of conjunctivitis or keratitis can only be evaluated after all contributing factors, like blinking rate, tear production, and tear fluid–drug interactions, are properly studied. Nevertheless, the reported results demonstrate that multiple antibiotics with low water solubility can be incorporated into a single formulation, which is convenient for both the patient and the physician in clinical practice, given, that the antimicrobial spectrum is increased and the number of daily treatments is decreased, as the in situ gel provides sustained release of the drugs, compared to conventional eye drops.

## 5. Conclusions

Antimicrobial resistance is a global problem in all medical fields, which encourages researchers to address this problem by increasing the effectiveness of antimicrobial products. In the case of ophthalmic preparations, in situ gels offer longer corneal retention time, more convenient application, and usually increased bioavailability compared to traditional eye drops. Thus, we aimed to formulate an in situ gel loaded with Azithromycin and Ofloxacin, two non-water-soluble antibiotics with different mechanisms of action. The novelty of our research is the combined use of the two molecules in a single preparation and their increased water solubility due to the proper concentration of poloxamer P188 and P407. Through full-box experimental design, the optimal ratio of the two polymers was measured, considering the physiological pH tolerance and the required relative viscosity. In the selected formulation, the water solubility of AZM was 1.125 mg/g and 1.25 mg/g in the case of OFX, which can be considered high values, compared to the other literature data. Our experiments verified that the formulation is not only able to dissolve the active agents but is capable of releasing the drugs from the gel matrix and the rheology of the formulation is temperature dependent, being a droppable Newtonian fluid at room temperature but forming a gel at the temperature of the ocular surface. Moreover, in vitro antimicrobial effectiveness was studied on actual clinical isolates of *S. aureus*, a relevant pathogen in case of eye infections. Our formulation could even prevent the growth of AZM- and OFX-resistant strains; thus, the combination proved to be effective in vitro. In order to prove clinical efficacy, the investigation of the long-term stability of the two antibiotics and in vivo bioavailability testing are needed. Both instability during storage and negative interaction with the tear fluid or quick elimination can severely limit the use of the formulation and such issues must be addressed immediately. However, the current stage of the research proved the increased solubility of the drugs, their advantageous antimicrobial interaction, and the optimal rheological behavior of the gel.

## Figures and Tables

**Table 1:** The quantities of poloxamer 407 and poloxamer 188.

Standard Run	Poloxamer 407 g/100 g Purified Water	Poloxamer 188 g/100 g Purified Water
R1	5	1
R2	5	3
R3	5	5
R4	15	1
R5	15	3
R6	15	5
R7	25	1
R8	25	3
R9	25	5

**Table 2:** The composition of the in situ gel formulation.

Composition of the Loaded Gel	Amount
Azithromycin	112.5 mg
Ofloxacin	125 mg
Poloxamer 407	20 g
Poloxamer 188	5 g
Purified Water	100 g

**Table 3:** The osmolality of gels was measured by a vapor pressure osmometer at (25 °C), n = 4. The means are calculated from four independent parallel samples.

Sample Name	Osmolality (mosmol/kg)
Unloaded gel	228 ± 1.1
Loaded gel	210 ± 1.0

## Data Availability

The original contributions presented in the study are included in the article, further inquiries can be directed to the corresponding author.
